# Editorial

**Published:** 1912-07

**Authors:** 


### EDITORIAL

The Business Office of the *Journal-Record* is 1 1-2 to 5 1-2 South Broad Street.
The Editorial Office is 1014-15 Century Building.
Address all Business Communications to *Journal-Record of Medicine*, 1 1-2 to 5 1-2 South Broad Street.
Make remittances payable *to The Journal-Record of Medicine.*

On matters pertaining to the Editorial and Original Communications, address Edgar G. Ballenger, M. D., Atlanta, Ga.
Reprints of Original Articles will be furnished at cost price. Requests for the same should always be made in THE MANUSCRIPT.

We will present, postpaid, on request, to each contributor of an original article, twenty (20) marked copies of *The Journal-Record of Medicine* containing such article.

### THE CHATTAHOOCHEE VALLEY MEDICAL AND SURGICAL ASSOCIATION.

This live and aggressive body of physicians met in Atlanta on July 16th and 17th, and a splendid meeting it was. A larger number of out-of-town members than usual were in attendance, which was pleasing indeed to the Atlanta medicos.

The program committee, under the persevering Chairman, Dr. Hansell Crenshaw, furnished a program replete with scientific pabulum; the local reception, or "glad-hand committee," consisting of Drs. Lokey, Boland, Selman and Mason vied with each other in making the visiting friends welcome, while the special "entertainment envoys extraordinary and ambassadors plenipotentiary," Drs. Daly and Arch Elkin, representing the Fulton County Medical Society, did themselves proud and no mistake about it.

Our able Secretary, Dr. Love, was ubiquitous during the meeting, seeing that every detail was carried out with order and prcision; while Mrs. Drake, his first lieutenant kept the records in her usual efficient manner.

The only feature that dimmed the joy of the occasion was the critical illness and death of Mrs. E. G. Ballenger, which occurred while the meeting was in progress. Dr. Ballenger is one of our most faithful and valued members, and he has the sympathy of the whole Association in his hour of distress and bereavement.

Atlanta always welcomes such representative bodies of men, and the University Club, at whose elegant quarters the meetings were held, will always be gratefully remembered for many acts of courtesy received.

The C. V. Association has come and gone, but their impress remains, and they will always be welcome to the Gate City of the South.

G. M. N.

---

### THE STATE'S NEED OF MEDICAL INSPECTION OF SCHOOLS.

Every country has its proverbs, handing them down from generation to generation. Some are based on superstition, while others are full of truth. The one "Prevention is better than cure," is common to almost every country, but few have made use of its truth.

Plato says, "Medicine is the Science of Health," but since the time of Aescalapius, th doctor in most countries has been treated as a curative rather than a preventive agent.

We seem to realize the force of the foregoing in the face of some terrible epidemic, but as soon as it is conquered, we resume our lethargy. However, of late years there has been some change of attitude in many of the foreign countries, as well as in the United States. One result of this change is the Medical Inspection of School Children. This work was inaugurated in Belgium in 1874. It very rapidly spread to the surrounding countries, Germany, England and France soon taking it in as a part of their Civil Government. No step seems to have been taken in the United States until 1892, when the Boston (Mass.) City Council moved that an appropriation be made to begin this work. Nothing more was done until 1894, when the money was raised and about the same time New York City began the inspection of some of her schools.

Everywhere the work gave good results and city after city took it up, until at present a majority of the cities and some of the states have laws governing the work.

The city of Atlanta began the inspection of her schools some five years ago and every year since has enlarged her corps, until the work is now carried on by an Inspector, an Assistant, and four trained nurses for the white schools, and adequate provision for the colored. Two of the counties in South Georgia have had their schools inspcted under the management of the Rockefeller Foundation and this is about the extent of the work in Georgia.

One year ago the Fulton County Board of School Commissioners decided to institute Medical Inspection in the schools of the county. I was elected to this place and my duties somewhat ill-defined. Owing to the peculiar conditions existing in the rural schools of this and others of the Southern States, there was no beaten path to follow and I had to somewhat outline my own course of work. It took some little time to get the teachers interested, for to them the work was new. While many of the patrons of the schools naturally took kindly to any movement which was to ultimately benefit their children, there were some who looked on it as an infringement of their rights as parents to govern their children. The children, too, had to be often re-assured, since the rural child is, on account of his somewhat isolated position, more timid than the city child.

Bearing these facts in mind, I tried to pick out major defects —those that were either prohibiting the child in proper attendance at school or bade fair to stunt its mental or physical development. Minor effects were left until the system became more thoroughly established, at which time they could be righted. We used the most common system, with a few modifications. At the top of the blank is the grade, on the left-hand side, a space for the name and ag, and a tthe top a list of the most common defects found; if no defect is found in a child, this is left blank, but if one or more exists this is marked under proper heading. The original is filed at the school and a carbon copy filed at the office of the County School Commissioner. The parent is notified of any defect found and asked to have this looked after.

In examining twenty-five hundred children I made note of four hundred and fifty defectives, there being in many of these more than one defect. This is a rather low percentage as compared with other statistics, but when taken in the light of the standard I used and the fact that already three-fourths of that number have been treated, it can be seen that no little good has been done the schools.

The most common defects were in order named: Defective Eyes, Tonsils and Adenoids; Anaemia and Defective Teeth. The teachers have reported that those children receiving treatment have, with few exceptions, been doing better work.

Also it has been my function to detect and isolate cases of infectious or contagious diseases; to look after the general hygiene and sanitation of the schools, and to instruct both teachers and pupils in these subjects.

Our right to conduct this work has come from some parts. If a state can compel a child to have its mind trained, cannot the same body assist in the maintenance of its health? A child is sent to school and every care taken that no harmful influence be brought to bear on the child from the school and we insist that no child shall bring harm to other children of the school. One child with Lung Trouble or Intestinal Parasite lays a whole school liable, not to mention those cases of the contagious diseases when one child breaks up a whole school for perhaps weeks. The classification of children is made hard when a part of the class have some eye or throat trouble that keeps them away from half or when there incapacitates them for proper application to work.

The justification of the state in assuming the function of education and making that education compulsory, is to insure its own preservation and efficiency. Whether or not it is successful will depend on its individual members. It is just as necessary that the proper physical laws shall be made, as the proper course of study prescribed. Not only our unwillingness to be outdone by foreign countries as well as other section of our own country, but our solicitude for our children will incite us to the incorporation of Medical Inspection as a part of our School System.

---

### MUNICIPAL HYGIENE.

A minister of the Gospel was once asked why his people were not more aroused to their danger in falling from grace as he placed it before them in his sermons. He said that they had hell and damnation preached at them so much that they were calloused and paid no attention to it. They were interested in other matters and attracted to worldly matters with changing programs.

It is a good deal so with Municipal Hygiene. We are used to people dying from typhoid fever in far larger numbers than should have the disease at all. We look with the unconcern of familiarity upon the long death list of tuberculosis. We note the ravages of malaria more because the disease has irregularities in its progress than on account of its destructiveness. And when we are told again and again that these great death dealing diseases are unnecessary and within our control if we but change our conduct in some slight degree, we feel as the congregation did and let hell and damnation go till another time. We have city and village development on other lines in our minds, and if some of the people are so careless or unfortunate as to get any of these diseases, it is their affair and they must suffer the consequences.

The occasion for this somewhat pessimistic and cold-blooded statement is the seemingly unrestrained presence of the fly and the lamentable lack of care men exercise in public spitting. In many communities, too, the mosquito is allowed to carry on its deadly progress without hindrance. We men of the Medical Profession know that the fly and the mosquito can be driven from any town and kept out together with the diseases they carry at the expense of well directed effort not half so great as that used in making improvements that will increase the market values of property or possibly add to their convenience.

We know that tuberculosis can be brought under control and eventually driven from the land by the exercise of hygienic precautions against its dissemination. Far more than half the deaths before 65 are due to tuberculosis, typhoid and malarial fevers. Practically all of these are preventable by correctly managed municipal hygiene.

We well know that sumptuary laws are of no avail where there is no definite public sentiment back of them to sustain them and insure the penalty of their disobedience. There is more to do than to make laws. The people must be taught in ways that will interest them that homes and villages and cities must be clean; clean not in the old-fashioned way, but in the new, intelligent, well-instructed way that keeps out disease and death as well as beautifies the locality. There should be no whited sepulchres; cleanliness must be complete and all-pervasive. Back yards could be as attractive as front lawns and roadways as tidy as porch floors. The labor is little and the reward is large.

The great potent element in bringing about this advantageous change is the educational influence of the family doctor. He can do more to convince his people than anyone else; he can find concrete instances in their family lives with which to illustrate his teachings and make the lessons clear. It devolves upon him, upon all of us therefore, to take up the life-saving work of sanitation and to make Municipal Hygiene an established fact.

